# Adjunctive gabexate mesylate with octreotide and clinical outcomes in older adults with acute pancreatitis: a retrospective study

**DOI:** 10.3389/fphar.2026.1755737

**Published:** 2026-10-14

**Authors:** Chao Zhu, Hua Feng, Liang Xu, Chun-Hong Li, Xiao-Hui Liu

**Affiliations:** 1 Department of Emergency, Qinhuangdao First Hospital, Qinhuangdao, China; 2 Department of Emergency, Qinhuangdao Third Hospital, Qinhuangdao, China; 3 Department of Emergency, Handan Hospital of Integrated Traditional Chinese and Western Medicine, Handan, China

**Keywords:** acute pancreatitis, clinical efficacy, elderly patients, gabexate mesylate, octreotide, systemic inflammatory response syndrome

## Abstract

**Background:**

Acute pancreatitis (AP) in older adults is associated with higher risks of systemic inflammatory response, organ dysfunction, and prolonged clinical recovery. Pharmacologic interventions targeting early protease activation and exocrine pancreatic secretion may have therapeutic value in this population; however, evidence in elderly patients remains limited.

**Methods:**

This retrospective study included 220 adults aged ≥60 years hospitalized with AP between July 2021 and July 2025. Among them, 108 received octreotide plus gabexate mesylate and 112 received octreotide monotherapy. Primary outcomes were times to abdominal pain relief, pancreatic enzyme normalization, and gastrointestinal recovery. Multiple linear and multivariable logistic regression models were used to adjust for prespecified demographic and clinical covariates.

**Results:**

Combination therapy was associated with shorter times to abdominal pain relief (adjusted mean difference −0.95 days; 95% confidence interval [CI] −1.28 to −0.62), pancreatic enzyme normalization (−1.28 days; 95% CI, −1.67 to −0.89), gastrointestinal recovery (−0.86 days; 95% CI −1.25 to −0.47), and hospital discharge (−1.62 days; 95% CI, −2.47 to −0.77; all P ≤ 0.001). It was also associated with higher odds of radiological improvement among re-imaged patients (adjusted odds ratio [OR], 1.82; 95% CI, 1.16–2.86) and lower odds of new-onset or persistent systemic inflammatory response syndrome (adjusted OR, 0.61; 95% CI, 0.38–0.98) and infectious complications (adjusted OR, 0.54; 95% CI, 0.29–0.99). Drug-related adverse events, composite organ dysfunction, mortality, and readmission did not differ significantly. No treatment-by-severity interaction was identified.

**Conclusion:**

Adjunctive gabexate mesylate was associated with modestly faster short-term recovery in older adults with acute pancreatitis, without a significant increase in drug-related adverse events. Its effects on organ dysfunction and mortality remain uncertain and require confirmation in prospective randomized studies.

## 1 Introduction

Acute pancreatitis (AP) is a common cause of emergency hospitalization and short-term morbidity, with rising admissions among older adults who experience disproportionate risks of organ dysfunction, infectious complications, and prolonged recovery. Age-related physiological reserve reduction, multimorbidity, and frailty contribute to adverse trajectories and higher resource utilization in this population (Beij et al., 2025; Wang et al., 2016). Recent cohort analyses demonstrate that frailty in AP is associated with increased local and systemic complications, greater healthcare use, and higher mortality, underscoring the need for targeted strategies that shorten the acute phase without adding treatment-related toxicity (Farooq et al., 2024; Fabb et al., 2026). Current guidelines emphasize high-quality supportive care as the cornerstone of AP management: early but not overly aggressive intravenous fluid resuscitation, adequate analgesia, early oral or enteral nutrition, avoidance of routine prophylactic antibiotics, and timely etiologic interventions (Song and Lee, 2024; Costea et al., 2025). Despite extensive investigation, no pharmacologic agent has been universally endorsed to alter the natural history of AP in unselected patients (Tenner et al., 2024; Yasunaga et al., 2013). State-of-the-art reviews from 2024 and contemporary society guidelines reaffirm that disease-modifying drugs remain investigational and that outcomes are largely driven by early recognition, severity stratification, and protocolized supportive measures (Nascimento et al., 2025).

A key early mechanistic event in AP is intrapancreatic activation of digestive proteases with downstream amplification via kallikrein–kinin and complement cascades, propagating systemic inflammatory response and microcirculatory injury. Gabexate mesylate, a broad-spectrum serine-protease inhibitor, can inhibit trypsin, kallikrein, and plasmin, thereby theoretically limiting autodigestion and mediator spillover (Zou et al., 2025). Historical randomized trials of gabexate in general AP populations yielded inconsistent clinical effects, contributing to skepticism about routine use; however, these trials were not designed to evaluate elderly-enriched cohorts or co-administration strategies that might augment pharmacodynamic impact (Moggia et al., 2017; Seta et al., 2014). Previous studies of protease inhibitors in acute pancreatitis have largely focused on heterogeneous adult populations and major outcomes such as mortality and severe complications, with inconsistent conclusions regarding clinical benefit (Seta et al., 2014; Abu-El-Haija et al., 2018). Evidence remains particularly limited in older adults, who may have distinct comorbidity profiles, reduced physiological reserve, and greater vulnerability to prolonged fasting, immobility, and hospitalization (Izquierdo et al., 2021; Qiao et al., 2025). Moreover, it has not been adequately established whether adjunctive gabexate mesylate combined with octreotide is associated with earlier clinical recovery than octreotide alone after accounting for disease severity, comorbidities, and treatment timing, or whether such associations differ across revised Atlanta severity categories (Bannone et al., 2023; Xie and Li, 2021).

To address this gap, we conducted a retrospective study of older adults with acute pancreatitis to evaluate the adjusted associations of combination therapy with short-term clinical recovery, complication-related outcomes, and healthcare utilization, together with exploratory severity-stratified and post-discharge analyses.

## 2 Methods

### 2.1 Study design

This retrospective study included elderly patients with a confirmed diagnosis of acute pancreatitis who received inpatient treatment at our institution between July 2021 and July 2025. Eligible patients were retrospectively categorized according to the treatment regimen documented in their medication records: the combination-therapy group received octreotide plus gabexate mesylate, whereas the monotherapy group received octreotide alone. Treatment selection occurred as part of routine clinical practice and was not assigned by the investigators. No randomization or prespecified institutional allocation algorithm was used. The attending physician selected the treatment regimen after discussion with the patient or family. The inclusion criteria were: (1) age ≥60 years; (2) a diagnosis of acute pancreatitis established according to internationally accepted diagnostic criteria based on compatible clinical manifestations, elevated serum pancreatic enzyme levels, and/or characteristic imaging findings; and (3) availability of sufficient baseline and in-hospital clinical data for the primary analyses. The exclusion criteria were: (1) chronic or recurrent pancreatitis; (2) severe pre-existing hepatic, renal, or cardiopulmonary dysfunction; (3) malignant tumors; (4) autoimmune pancreatitis; and (5) insufficient baseline or primary-outcome data. Informed consent was obtained from all participants. The study was reviewed and approved by the hospital’s ethics committee and conducted in accordance with relevant guidelines and the Declaration of Helsinki. Data confidentiality was maintained, with personal identifiers removed prior to analysis to ensure participant privacy.

### 2.2 Treatment protocol

All patients received supportive management for acute pancreatitis according to routine institutional practice. Supportive treatment included fasting when clinically indicated, gastrointestinal decompression, fluid resuscitation, electrolyte correction, analgesic therapy, glucose management, nutritional support according to disease severity and gastrointestinal tolerance, and surveillance for systemic and local complications. Hemodynamic parameters, urine output, serum electrolytes, pancreatic enzymes, inflammatory biomarkers, and organ function indices were monitored during hospitalization. Patients in the combination-therapy group received octreotide at a dose of 0.1 mg intravenously three times daily using an infusion pump. Gabexate mesylate was additionally administered by continuous intravenous infusion at a total daily dose of 1.5–2.0 g diluted in 250–500 mL of normal saline over 24 h. The gabexate mesylate dose was determined according to clinical tolerance, hemodynamic status, and renal function. Patients in the monotherapy group received octreotide alone using the same dose and administration schedule. No other protease inhibitor was administered in the monotherapy group. Treatment duration was determined by the attending physician according to the patient’s clinical course, including abdominal pain, gastrointestinal function, serum amylase and lipase trends, inflammatory markers, and overall clinical stability. Gabexate mesylate was used as an adjunctive treatment selected during routine clinical practice and was not considered a guideline-mandated standard treatment. Prophylactic antibiotics were not routinely administered. Antibiotic treatment was initiated when bacterial infection was clinically suspected or documented on the basis of microbiological findings, persistently elevated inflammatory markers, or imaging evidence of infectious complications. Mechanical ventilation, vasoactive drugs, renal replacement therapy, and interventional, endoscopic, or surgical procedures were provided when clinically indicated.

### 2.3 Data collection and outcome definitions

Clinical data were retrospectively abstracted from the institutional electronic medical record. Two trained reviewers used a predefined data-extraction codebook. Disagreements were resolved through review by a senior clinician. A randomly selected 10% sample of records was audited for accuracy and completeness. Recorded baseline characteristics included age, sex, body mass index, hypertension, diabetes mellitus, coronary artery disease, chronic kidney disease, and acute pancreatitis etiology. Etiology was classified as biliary, hypertriglyceridemia-related, alcohol-related, or idiopathic according to physician documentation supported by laboratory findings, biliary imaging, and available endoscopic or operative records. Acute pancreatitis severity was classified as mild, moderately severe, or severe according to the revised Atlanta classification. Vital signs and clinical data recorded during the first 24 h of hospitalization were used to determine the Acute Physiology and Chronic Health Evaluation II score. Systemic inflammatory response syndrome status at admission was also recorded. Admission laboratory measurements included C-reactive protein, procalcitonin, serum amylase, serum lipase, blood urea nitrogen, serum creatinine, white blood cell count, liver enzymes, total bilirubin, electrolytes, glucose, and coagulation indices. For repeatedly measured variables, the clinically relevant peak, lowest, or worst value was recorded according to the prespecified data-extraction protocol. The time from symptom onset to treatment initiation was calculated in hours using the patient-reported time of symptom onset corroborated, when possible, by triage and admission documentation. Medication route, dose, start and stop times, and treatment duration were extracted from medication records. Co-interventions, including fluid therapy, analgesia, nutritional support, antibiotics, mechanical ventilation, vasopressor therapy, and renal replacement therapy, were also recorded.

#### 2.3.1 Primary outcomes

The primary outcomes were time from treatment initiation to abdominal pain relief, normalization of serum pancreatic enzymes, and recovery of gastrointestinal function. Abdominal pain relief was defined as the first documented sustained clinical improvement in abdominal pain without the need for escalation of analgesic treatment. Normalization of serum pancreatic enzymes was defined as the first documented return of serum amylase and/or lipase to the institutional reference range or a sustained decline considered by the treating team to be consistent with biochemical remission. Recovery of gastrointestinal function was defined as the documented return of bowel motility and tolerance of oral intake, together with resolution of clinically significant abdominal distension and discontinuation of gastrointestinal decompression when previously required.

#### 2.3.2 Secondary clinical outcomes

Secondary outcomes included length of hospital stay, duration of bowel rest, duration of intravenous medication infusion, antibiotic requirement, intensive care unit admission, radiological improvement, and necrotic progression. Length of hospital stay was calculated from admission to discharge. Duration of bowel rest was calculated from treatment initiation to resumption of oral or enteral intake. Duration of intravenous medication infusion was calculated from initiation to discontinuation of intravenous treatment. Antibiotic requirement referred to antibiotic treatment initiated during the index hospitalization for suspected or confirmed infection. Intensive care unit admission was defined as transfer to or treatment in the intensive care unit during the index hospitalization. Baseline and clinically indicated follow-up ultrasonography, contrast-enhanced computed tomography, or magnetic resonance imaging were reviewed by an attending radiologist blinded to treatment group. Follow-up imaging was not performed according to a standardized study schedule and was obtained only when clinically indicated. Radiological improvement was defined as a reduction in pancreatic edema or peripancreatic fluid collections without progression of pancreatic necrosis on repeat imaging. Necrotic progression was defined as a new or increased area of pancreatic or peripancreatic necrosis on follow-up imaging. Radiological outcomes were evaluated only among patients who underwent repeat imaging. Patients without follow-up imaging were not classified as having or not having radiological improvement or necrotic progression.

#### 2.3.3 Safety and complication outcomes

Safety and complication outcomes included new-onset or persistent systemic inflammatory response syndrome after treatment initiation, composite organ dysfunction, respiratory failure, renal impairment, circulatory instability, infectious complications, invasive supportive interventions, drug-related adverse events, and in-hospital mortality. Systemic inflammatory response syndrome was identified according to standard clinical criteria requiring at least two abnormalities involving body temperature, heart rate, respiratory rate or arterial carbon dioxide tension, and white blood cell count. New-onset systemic inflammatory response syndrome referred to systemic inflammatory response syndrome first documented after treatment initiation among patients without systemic inflammatory response syndrome at admission. Persistent systemic inflammatory response syndrome referred to continued fulfillment of systemic inflammatory response syndrome criteria after treatment initiation among patients with systemic inflammatory response syndrome at admission. Composite organ dysfunction was defined as the occurrence of at least one of respiratory failure, renal impairment, or circulatory instability during hospitalization. Infectious complications included clinically documented infections requiring antimicrobial treatment. Invasive supportive interventions included mechanical ventilation, vasopressor treatment, or renal replacement therapy. Drug-related adverse events were identified from physician and nursing documentation and included infusion-related reactions, transient hypotension, and other events considered possibly or probably related to octreotide or gabexate mesylate. In-hospital mortality was defined as death from any cause during the index hospitalization.

#### 2.3.4 Exploratory post-discharge outcomes

Exploratory post-discharge outcomes included 30-day and 90-day all-cause mortality calculated from the date of the index admission and 30-day all-cause institutional readmission calculated from hospital discharge. Mortality status was ascertained from institutional electronic medical records and available follow-up documentation. Institutional readmission included only readmissions recorded at the study institution. The 30-day readmission analysis was restricted to patients who were discharged alive, remained alive at 30 days, and had ascertainable readmission status. Patients without ascertainable follow-up information were excluded from the corresponding analysis.

### 2.4 Statistical analysis

Statistical analyses were performed using SPSS version 28.0 (IBM Corp., Armonk, NY, USA). Before analysis, range checks, logical consistency checks, and chronological checks were performed to identify implausible or internally inconsistent values. Continuous variables were summarized as mean ± standard deviation and compared using Welch’s t-test or the Mann–Whitney U test, as appropriate. Categorical variables were presented as frequencies and percentages and compared using Pearson’s chi-square test or Fisher’s exact test. Absolute standardized mean differences were calculated to assess baseline imbalance, with values > 0.10 considered potentially meaningful. Unadjusted effects were expressed as mean differences or odds ratios (ORs) with 95% confidence intervals (CIs). Multiple linear regression was used to estimate adjusted mean differences for continuous outcomes, whereas multivariable logistic regression was used to estimate adjusted ORs for binary outcomes. Covariates were selected *a priori* according to clinical relevance and included demographic characteristics, comorbidities, biliary etiology, APACHE II score, SIRS at admission, revised Atlanta severity where applicable, and time from symptom onset to treatment initiation. Radiological analyses were restricted to patients who underwent repeat imaging. Effect modification by acute pancreatitis severity was assessed using treatment-by-severity interaction terms. Firth penalized logistic regression was used for the exploratory mortality and readmission analyses because of the small number of events. Multicollinearity and model assumptions were assessed using variance inflation factors, Breusch–Pagan tests, Q–Q plots, Hosmer–Lemeshow tests, and receiver operating characteristic curves, as appropriate. Baseline covariates with <5% missingness were imputed using the median or mode; outcomes were not imputed. All tests were two-sided, with P < 0.05 considered statistically significant.

## 3 Results

### 3.1 Baseline characteristics

A total of 220 older adults with acute pancreatitis were included, comprising 108 patients who received octreotide plus gabexate mesylate and 112 who received octreotide monotherapy. No statistically significant between-group differences were observed in age, sex, body mass index, comorbidities, acute pancreatitis etiology, revised Atlanta severity, APACHE II score, SIRS on admission, or time from symptom onset to treatment initiation. However, the absolute standardized mean differences indicated modest imbalances in several variables, including biliary etiology, age, APACHE II score, and time to treatment initiation. The proportions of patients with mild, moderately severe, and severe acute pancreatitis were 49.1%, 35.2%, and 15.7% in the combination-therapy group and 55.4%, 31.3%, and 13.4% in the monotherapy group, respectively (Table 1). Additional baseline laboratory characteristics are presented in Supplementary Table S1.

**TABLE 1:** Selected baseline characteristics of elderly patients with acute pancreatitis.

Variable	Combination therapy (n = 108)	Octreotide monotherapy (n = 112)	P value	Absolute SMD
Age, years	71.9 ± 5.5	73.1 ± 5.7	0.113	0.214
Male sex, n (%)	60 (55.6)	70 (62.5)	0.295	0.142
Body mass index, kg/m^2^	24.6 ± 3.4	24.7 ± 3.2	0.823	0.030
Hypertension, n (%)	53 (49.1)	61 (54.5)	0.424	0.108
Diabetes mellitus, n (%)	35 (32.4)	27 (24.1)	0.171	0.185
Coronary artery disease, n (%)	10 (9.3)	17 (15.2)	0.181	0.181
Chronic kidney disease, n (%)	13 (12.0)	8 (7.1)	0.217	0.167
Etiology, global P value	​	​	0.205	—
Biliary	64 (59.3)	51 (45.5)	—	0.277
Hypertriglyceridemia	21 (19.4)	31 (27.7)	—	0.195
Alcohol-related	7 (6.5)	7 (6.3)	—	0.009
Idiopathic	16 (14.8)	23 (20.5)	—	0.150
Revised Atlanta severity, global P value	​	​	0.644	—
Mild	53 (49.1)	62 (55.4)	—	0.126
Moderately severe	38 (35.2)	35 (31.3)	—	0.084
Severe	17 (15.7)	15 (13.4)	—	0.067
APACHE II score	9.7 ± 2.8	9.1 ± 2.9	0.120	0.210
SIRS on admission, n (%)	48 (44.4)	55 (49.1)	0.488	0.094
Time from symptom onset to treatment initiation, h	19.7 ± 8.0	17.8 ± 8.6	0.091	0.229

Abbreviations: APACHE II, Acute Physiology and Chronic Health Evaluation II; SIRS, systemic inflammatory response syndrome; SMD, standardized mean difference.

### 3.2 Primary clinical outcomes

The mean time to abdominal pain relief was 3.2 ± 1.1 days in the combination-therapy group and 4.1 ± 1.3 days in the monotherapy group, corresponding to an unadjusted mean difference of −0.90 days (95% CI, −1.22 to −0.58; P < 0.001). The corresponding mean differences were −1.20 days for normalization of serum pancreatic enzymes (95% CI, −1.67 to −0.73; P < 0.001) and −0.90 days for recovery of gastrointestinal function (95% CI, −1.30 to −0.50; P < 0.001) (Table 2).

**TABLE 2:** Primary efficacy outcomes in elderly patients with acute pancreatitis.

Outcome	Combination therapy (n = 108)	Octreotide monotherapy (n = 112)	Unadjusted mean difference, days	95% CI	P value
Time to abdominal pain relief	3.2 ± 1.1	4.1 ± 1.3	−0.90	−1.22 to −0.58	<0.001
Time to normalization of serum pancreatic enzymes	4.9 ± 1.6	6.1 ± 1.9	−1.20	−1.67 to −0.73	<0.001
Time to recovery of gastrointestinal function	3.6 ± 1.4	4.5 ± 1.6	−0.90	−1.30 to −0.50	<0.001

Abbreviation: CI, confidence interval.

### 3.3 Secondary clinical outcomes

The combination-therapy group had a shorter mean hospital stay than the monotherapy group (9.1 ± 3.2 vs. 11.0 ± 3.8 days; mean difference, −1.90 days; 95% CI, −2.83 to −0.97; P < 0.001). The mean differences were −0.80 days for bowel rest (95% CI, −1.25 to −0.35; P = 0.001) and −0.70 days for intravenous medication infusion (95% CI, −1.18 to −0.22; P = 0.004). Antibiotics were required by 25.9% of patients in the combination-therapy group and 38.4% in the monotherapy group (OR, 0.56; 95% CI, 0.32–1.00; P = 0.048). ICU admission occurred in 11.1% and 19.6% of patients, respectively (OR, 0.51; 95% CI, 0.24–1.09; P = 0.080). Among patients who underwent clinically indicated repeat imaging, radiological improvement was recorded in 79.1% of the combination-therapy group and 62.2% of the monotherapy group (OR, 2.29; 95% CI, 1.17–4.49; P = 0.014) (Table 3).

**TABLE 3:** Secondary clinical outcomes in elderly patients with acute pancreatitis.

Outcome	Combination therapy	Octreotide monotherapy	Unadjusted effect estimate	95% CI	P value
Length of hospital stay, days	9.1 ± 3.2	11.0 ± 3.8	MD −1.90	−2.83 to −0.97	<0.001
Duration of bowel rest, days	3.9 ± 1.6	4.7 ± 1.8	MD −0.80	−1.25 to −0.35	0.001
Duration of intravenous medication infusion, days	4.6 ± 1.7	5.3 ± 1.9	MD −0.70	−1.18 to −0.22	0.004
Antibiotic requirement, n (%)	28/108 (25.9)	43/112 (38.4)	OR 0.562	0.316 to 0.998	0.048
ICU admission, n (%)	12/108 (11.1)	22/112 (19.6)	OR 0.51	0.24 to 1.09	0.080
Radiological improvement, n/re-imaged (%)	68/86 (79.1)	56/90 (62.2)	OR 2.29	1.17 to 4.49	0.014
Necrotic progression, n/re-imaged (%)	9/86 (10.5)	18/90 (20.0)	OR 0.47	0.20 to 1.11	0.079

Abbreviations: CI, confidence interval; ICU, intensive care unit; MD, mean difference; OR, odds ratio.

### 3.4 Safety and complication outcomes

New-onset or persistent SIRS after treatment initiation occurred in 27.8% of patients in the combination-therapy group and 41.1% in the monotherapy group (OR, 0.55; 95% CI, 0.31–0.97; P = 0.038). No statistically significant between-group differences were observed in composite organ dysfunction, infectious complications, in-hospital mortality, or drug-related adverse events (Table 4). Organ-specific complications and invasive supportive interventions are reported in Supplementary Table S2.

**TABLE 4:** Core safety outcomes in elderly patients with acute pancreatitis.

Outcome	Combination therapy (n = 108)	Octreotide monotherapy (n = 112)	Unadjusted OR	95% CI	P value
New-onset or persistent SIRS after treatment initiation	30 (27.8)	46 (41.1)	0.55	0.31 to 0.97	0.038
Composite organ dysfunction	19 (17.6)	30 (26.8)	0.58	0.31 to 1.12	0.101
Infectious complications	15 (13.9)	26 (23.2)	0.53	0.26 to 1.07	0.076
In-hospital mortality	3 (2.8)	6 (5.4)	0.5	0.12 to 2.07	0.499
Drug-related adverse events	6 (5.6)	5 (4.5)	1.26	0.37 to 4.25	0.765

Abbreviations: CI, confidence interval; OR, odds ratio; SIRS, systemic inflammatory response syndrome.

^P^ values were calculated using Pearson’s chi-square test, except for in-hospital mortality and drug-related adverse events, for which Fisher’s exact test was used.

### 3.5 Adjusted associations between treatment regimen and clinical outcomes

After adjustment for prespecified demographic and clinical covariates, combination therapy was associated with shorter times to abdominal pain relief (adjusted mean difference, −0.95 days; 95% CI, −1.28 to −0.62; P < 0.001), normalization of serum pancreatic enzymes (−1.28 days; 95% CI, −1.67 to −0.89; P < 0.001), and recovery of gastrointestinal function (−0.86 days; 95% CI, −1.25 to −0.47; P < 0.001).

The adjusted mean differences were −1.62 days for length of hospital stay (95% CI, −2.47 to −0.77; P < 0.001), −0.73 days for bowel rest (95% CI, −1.17 to −0.29; P = 0.001), and −0.58 days for intravenous medication infusion (95% CI, −1.04 to −0.12; P = 0.014).

Among the 176 patients who underwent repeat imaging, combination therapy was associated with higher odds of radiological improvement (adjusted OR, 1.82; 95% CI, 1.16–2.86; P = 0.009). In the full cohort, it was associated with lower odds of new-onset or persistent SIRS (adjusted OR, 0.61; 95% CI, 0.38–0.98; P = 0.041) and infectious complications (adjusted OR, 0.54; 95% CI, 0.29–0.99; P = 0.047). The association with composite organ dysfunction was not statistically significant (adjusted OR, 0.68; 95% CI, 0.37–1.24; P = 0.209) (Table 5). Model-specific covariates and analysis populations are detailed in Supplementary Table S3, and model diagnostics are provided in Supplementary Table S4.

**TABLE 5:** Adjusted associations between treatment regimen and selected clinical outcomes.

Outcome	Analysis population	Adjusted mean difference or	95% CI	P value
Continuous outcomes: Adjusted mean difference, days				
Time to abdominal pain relief	n = 220	−0.95	−1.28 to −0.62	<0.001
Time to normalization of serum pancreatic enzymes	n = 220	−1.28	−1.67 to −0.89	<0.001
Time to recovery of gastrointestinal function	n = 220	−0.86	−1.25 to −0.47	<0.001
Length of hospital stay	n = 220	−1.62	−2.47 to −0.77	<0.001
Duration of bowel rest	n = 220	−0.73	−1.17 to −0.29	0.001
Duration of intravenous medication infusion	n = 220	−0.58	−1.04 to −0.12	0.014
Binary outcomes: Adjusted OR				
Radiological improvement	n = 176; 124 events	1.82	1.16 to 2.86	0.009
New-onset or persistent SIRS after treatment initiation	n = 220; 76 events	0.61	0.38 to 0.98	0.041
Infectious complications	n = 220; 41 events	0.54	0.29 to 0.99	0.047
Composite organ dysfunction	n = 220; 49 events	0.68	0.37 to 1.24	0.209

Continuous outcomes were analyzed using multiple linear regression and are presented as adjusted mean differences. Binary outcomes were analyzed using multivariable logistic regression and are presented as adjusted ORs. Octreotide monotherapy was the reference group. Model-specific covariates and analysis populations are provided in (Supplementary Table S4).

Abbreviations: CI, confidence interval; OR, odds ratio; SIRS, systemic inflammatory response syndrome.

### 3.6 Exploratory subgroup analyses according to the revised atlanta classification

The adjusted associations for the continuous outcomes were directionally consistent across the mild, moderately severe, and severe acute pancreatitis subgroups. No statistically significant treatment-by-severity interactions were observed for abdominal pain relief (P for interaction = 0.83), normalization of serum pancreatic enzymes (P for interaction = 0.79), recovery of gastrointestinal function (P for interaction = 0.87), or length of hospital stay (P for interaction = 0.61). Estimates in the severe subgroup had wider confidence intervals because this subgroup included only 32 patients (Supplementary Table S5).

### 3.7 Exploratory analyses of 30- and 90-day mortality and readmission

Thirty-day all-cause mortality was 3.7% in the combination-therapy group and 7.1% in the monotherapy group (Firth-adjusted OR, 0.52; 95% CI, 0.14–1.92). Ninety-day all-cause mortality was 5.6% and 9.8%, respectively (Firth-adjusted OR, 0.58; 95% CI, 0.20–1.68). Among patients discharged alive, alive at 30 days, and with ascertainable readmission status, 30-day institutional readmission occurred in 9.7% of the combination-therapy group and 16.3% of the monotherapy group (Firth-adjusted OR, 0.59; 95% CI, 0.25–1.39). The confidence intervals for these outcomes included the null value (Supplementary Table S6).

## 4 Discussion

In this retrospective cohort of older adults with acute pancreatitis, adjunctive gabexate mesylate combined with octreotide was associated with shorter times to abdominal pain relief, normalization of serum pancreatic enzymes, and recovery of gastrointestinal function compared with octreotide alone. Combination therapy was also associated with shorter durations of bowel rest, intravenous medication infusion, and hospitalization. Among patients who underwent clinically indicated repeat imaging, radiological improvement was more frequently observed in the combination-therapy group; however, this finding applies only to the selected subgroup that underwent radiological reassessment. The frequency of drug-related adverse events did not differ significantly between groups. New-onset or persistent SIRS and infectious complications were less frequent after multivariable adjustment, whereas the associations with composite organ dysfunction and mortality were not statistically significant. These findings represent observational associations and should not be interpreted as evidence of causal treatment effects.

The clinical interpretation of these findings depends not only on statistical significance but also on the magnitude and relevance of the observed differences. The adjusted reductions in time to symptom relief, biochemical normalization, gastrointestinal recovery, and hospital discharge were generally modest, indicating that adjunctive gabexate mesylate was associated with an earlier recovery trajectory rather than a large change in the overall course of acute pancreatitis. Among these outcomes, relief of abdominal pain and restoration of gastrointestinal function may be more directly relevant to patients because they affect comfort, oral intake, mobilization, and readiness for discharge. In contrast, normalization of pancreatic enzymes should be regarded primarily as a biochemical indicator, as serum amylase and lipase levels do not necessarily reflect the extent of pancreatic injury or predict organ failure. The concurrent shortening of bowel rest, intravenous treatment, and hospitalization nevertheless provides internal consistency across several related dimensions of early recovery. This pattern may be particularly relevant in older adults, in whom prolonged fasting, immobility, and hospitalization can contribute to nutritional deterioration, functional decline, and delayed rehabilitation (Banfi et al., 2005; Pezzilli and Miglioli, 2001). However, differences of less than approximately one to 2 days should be interpreted in the context of the absence of established minimum clinically important differences for these outcomes (Kohsaki et al., 2004; Andriulli et al., 2003). Accordingly, the practical value of the observed associations may depend on whether they translate into meaningful reductions in treatment burden, resource utilization, or loss of functional independence. The results therefore support a possible role for adjunctive therapy in accelerating selected short-term recovery processes, but they do not by themselves demonstrate a substantial modification of disease severity or prognosis.

The overall pattern across complication-related outcomes suggests a distinction between markers of early inflammatory activity and more definitive measures of disease progression. The associations observed for SIRS and infectious complications may indicate that adjunctive treatment was linked to a more favorable early inflammatory course, whereas the absence of clear differences in organ dysfunction, ICU admission, necrotic progression, and mortality suggests that this pattern did not extend consistently to severe clinical endpoints. This hierarchy of findings is important because improvements in inflammatory or radiological markers should not be interpreted as equivalent to prevention of organ failure, pancreatic necrosis, or death. Similarly, the greater frequency of radiological improvement among patients who underwent reassessment may reflect concordance between clinical and local morphological recovery, but the lack of a corresponding association with necrotic progression argues against a strong effect on structural disease evolution. The severity-stratified analyses further showed broadly similar directions of association across revised Atlanta categories, with no evidence of statistically significant treatment-by-severity interaction. This indicates that the observed short-term associations were not clearly confined to a particular severity group, although it does not establish uniform effectiveness across all categories (Trikudanathan et al., 2024; Guilabert et al., 2025). The exploratory follow-up findings provide an additional perspective: numerically lower 30- and 90-day mortality and 30-day readmission rates were not accompanied by statistically conclusive estimates. Thus, the apparent advantages were concentrated in short-term recovery measures rather than in major post-discharge outcomes. Taken together, these results are most consistent with a potential adjunctive effect on early clinical recovery, while providing insufficient evidence that gabexate mesylate alters the risk of severe complications or longer-term prognosis. This interpretation is also more consistent with current management frameworks, which prioritize supportive care and do not consider protease inhibitors routine standard therapy (Hamesch et al., 2025; Sohail et al., 2024).

The clinical role of octreotide in acute pancreatitis remains uncertain, and most guidelines do not recommend routine use of somatostatin analogues, although practice patterns vary across regions. Earlier clinical studies reported potential reductions in selected complications, inflammatory activity, disease progression, and hospital stay with octreotide treatment (Paran et al., 2000; Yang et al., 2012; Wang et al., 2013), while experimental evidence has suggested possible effects on pancreatic secretion, pyroptosis, and intestinal homeostasis (Zhao et al., 2025). However, these findings have not established a consistent clinical benefit, particularly in older adults, who often have multiple comorbidities, reduced physiological reserve, and greater vulnerability to systemic inflammation, organ dysfunction, prolonged fasting, and hospitalization. Combination strategies involving somatostatin analogues and agents targeting inflammatory or protease-mediated pathways have therefore attracted interest as a means of addressing complementary components of acute pancreatitis pathophysiology (Sundar et al., 2020; Bertilsson and Kalaitzakis, 2015). In the present study, octreotide was administered in both groups and served as background therapy; the relevant treatment contrast was the addition of gabexate mesylate. Gabexate may theoretically complement octreotide through inhibition of protease activity and modulation of inflammatory responses, although the present observational study did not directly evaluate these mechanisms. Within routine supportive care, adjunctive gabexate mesylate was associated with modestly shorter times to pain relief, pancreatic enzyme normalization, gastrointestinal recovery, and hospital discharge. It was also associated with lower adjusted odds of systemic inflammatory response syndrome and infectious complications, whereas associations with organ dysfunction, mortality, and other major outcomes were not conclusive. Drug-related adverse events did not differ significantly between groups (Machicado and Papachristou, 2019; Hey-Hadavi et al., 2023). These findings therefore support further evaluation of gabexate as an adjunct to octreotide in older adults, but they should not be interpreted as evidence that combination therapy alters disease severity or long-term prognosis. Prospective randomized studies are needed to determine whether the observed short-term associations represent clinically meaningful treatment benefits (Singh and Gorelick, 2024).

Several limitations should be considered when interpreting these findings. First, the retrospective, single-center, and nonrandomized design introduces risks of selection bias and confounding by indication. Treatment was selected during routine clinical practice rather than according to a prespecified allocation protocol. Although multivariable models adjusted for measured demographic and clinical characteristics, residual and unmeasured confounding related to physician preference, frailty, supportive-care intensity, and subtle differences in baseline disease status cannot be excluded. Second, radiological reassessment was clinically indicated rather than protocol mandated; therefore, imaging outcomes were derived from a selected subgroup and may have been affected by selection and ascertainment bias. Third, the sample size limited the precision of estimates for uncommon outcomes, including mortality, organ-specific failure, and other major complications. Fourth, several statistically significant differences were modest in absolute magnitude, and established minimum clinically important differences are unavailable for pain relief, enzyme normalization, gastrointestinal recovery, and length of stay. These findings should therefore be interpreted in relation to patient-centered benefit and resource utilization rather than statistical significance alone. Fifth, readmissions to other institutions may not have been captured, and the available follow-up did not permit comprehensive assessment of recurrent pancreatitis, long-term functional recovery, quality of life, overall survival, or healthcare utilization. Despite these limitations, the study incorporated multivariable adjustment, severity-stratified analyses, predefined outcome definitions, and exploratory post-discharge assessments, providing a more detailed evaluation than unadjusted short-term comparisons alone. Future multicenter prospective studies should apply standardized treatment-allocation procedures, harmonized supportive-care protocols, and scheduled imaging assessments. Randomized controlled trials with larger and more diverse populations are needed to confirm efficacy and safety in both older and younger patients and to determine whether treatment effects vary by disease severity or age-related risk profile. Future studies should also evaluate optimal gabexate dosing, timing, treatment duration, cost-effectiveness, and longer-term outcomes, including persistent organ failure, infected necrosis, mortality, readmission, recurrence, functional recovery, and patient-reported outcomes.

## 5 Conclusions

In this retrospective cohort of older adults with acute pancreatitis, adjunctive gabexate mesylate combined with octreotide was associated with modestly shorter times to abdominal pain relief, pancreatic enzyme normalization, gastrointestinal recovery, and hospital discharge compared with octreotide alone. No statistically significant between-group difference was observed in drug-related adverse events, whereas associations with major outcomes, including organ dysfunction and mortality, were not conclusive. Prospective randomized studies are required to determine whether adjunctive gabexate mesylate provides clinically meaningful benefits.

## Data Availability

The raw data supporting the conclusions of this article will be made available by the authors, without undue reservation.

## References

[B1] Abu-El-Haija M. Kumar S. Quiros J. A. Balakrishnan K. Barth B. Bitton S. (2018). Management of acute pancreatitis in the pediatric population: a clinical report from the north American society for pediatric gastroenterology, hepatology and nutrition pancreas committee. J. Pediatr. Gastroenterol. Nutr. 66 (1), 159–176. 10.1097/mpg.0000000000001715 29280782 PMC5755713

[B2] Andriulli A. Caruso N. Quitadamo M. Forlano R. Leandro G. Spirito F. (2003). Antisecretory vs. antiproteasic drugs in the prevention of post-ERCP pancreatitis: the evidence-based medicine derived from a meta-analysis study. Jop 4 (1), 41–48. 12555015

[B3] Banfi R. Borselli G. Cappelletti S. Mari L. Aiazzi M. Taddei V. (2005). Gabexate mesilate and acute pancreatitis: an experience of evidence based drug information for improving rational drug use. Pharm. World Sci. 27 (2), 121–123. 10.1007/s11096-004-5692-4 15999923

[B4] Bannone E. Pulvirenti A. Marchegiani G. Vacca P. G. Marchetti A. Cattelani A. (2023). No role for protease inhibitors as a mitigation strategy for postpancreatectomy acute pancreatitis (PPAP): propensity score matching analysis. Pancreatology 23 (8), 904–910. 10.1016/j.pan.2023.09.142 37839921

[B5] Beij A. Verdonk R. C. van Santvoort H. C. de-Madaria E. Voermans R. P. (2025). Acute pancreatitis: an update of evidence-based management and recent trends in treatment strategies. United Eur. Gastroenterol. J. 13 (1), 97–106. 10.1002/ueg2.12743 39804691 PMC11866315

[B6] Bertilsson S. Kalaitzakis E. (2015). Acute pancreatitis and use of pancreatitis-associated drugs: a 10-Year population-based cohort study. Pancreas 44 (7), 1096–1104. 10.1097/mpa.0000000000000406 26335010

[B7] Costea C. N. Pojoga C. Seicean A. (2025). Advances in the management of fluid resuscitation in acute pancreatitis: a systematic review. Diagn. (Basel) 15 (7), 810. 10.3390/diagnostics15070810 40218161 PMC11988764

[B8] Fabbri E. Tebano G. de Angelis A. Del Prete A. Maestri L. Cristini F. (2026). Sepsis in frail older adults: tailored antimicrobial stewardship and individualized care approach. Antibiotics 15 (5), 496. 10.3390/antibiotics15050496 42192718 PMC13203186

[B9] Farooq U. Abbasi A. F. Tarar Z. I. Chaudhary A. J. Kamal F. (2024). Understanding the role of frailty in local and systemic complications and healthcare resource utilization in acute pancreatitis: findings from a national cohort. Pancreatology 24 (1), 6–13. 10.1016/j.pan.2023.12.001 38072685

[B10] Guilabert L. Cárdenas-Jaén K. de-Madaria E. (2025). Initial management of acute pancreatitis. Gastroenterol. Clin. North Am. 54 (1), 21–36. 10.1016/j.gtc.2024.07.001 39880529

[B11] Hamesch K. Hollenbach M. Guilabert L. Lahmer T. Koch A. (2025). Practical management of severe acute pancreatitis. Eur. J. Intern. Med. 133, 1–13. 10.1016/j.ejim.2024.10.030 39613703

[B12] Hey-Hadavi J. Velisetty P. Mhatre S. (2023). Trends and recent developments in pharmacotherapy of acute pancreatitis. Postgrad. Med. 135 (4), 334–344. 10.1080/00325481.2022.2136390 36305300

[B13] Izquierdo M. Merchant R. A. Morley J. E. Anker S. D. Aprahamian I. Arai H. (2021). International exercise recommendations in older adults (ICFSR): Expert consensus guidelines. Journal Nutrition, Health and Aging 25 (7), 824–853. 10.1007/s12603-021-1665-8 34409961 PMC12369211

[B14] Kohsaki T. Nishimori I. Onishi S. (2004). Treatment of acute pancreatitis with protease inhibitor, H2 receptor antagonist and somatostatin analogue. Nihon Rinsho 62 (11), 2057–2062. 15552889

[B15] Machicado J. D. Papachristou G. I. (2019). Pharmacologic management and prevention of acute pancreatitis. Curr. Opin. Gastroenterol. 35 (5), 460–467. 10.1097/mog.0000000000000563 31205053

[B16] Moggia E. Koti R. Belgaumkar A. P. Fazio F. Pereira S. P. Davidson B. R. (2017). Pharmacological interventions for acute pancreatitis. Cochrane Database Syst. Rev. 4 (4), Cd011384. 10.1002/14651858.CD011384.pub2 28431202 PMC6478067

[B17] Nascimento M. M. P. Azuelos A. Nóbrega I. L. P. Pitombeira M. S. Martinez A. T. A. de Carvalho J. F. (2025). State of the art in novel treatment strategies in rheumatoid arthritis: a brief review. Mediterr. J. Rheumatol. 36 (2), 149–158. 10.31138/mjr.241124.ath 40757117 PMC12312483

[B18] Paran H. Mayo A. Paran D. Neufeld D. Shwartz I. Zissin R. (2000). Octreotide treatment in patients with severe acute pancreatitis. Dig. Dis. Sci. 45 (11), 2247–2251. 10.1023/a:1026679106463 11215748

[B19] Pezzilli R. Miglioli M. (2001). Multicentre comparative study of two schedules of gabexate mesilate in the treatment of acute pancreatitis. Italian acute pancreatitis study group. Dig. Liver Dis. 33 (1), 49–57. 10.1016/s1590-8658(01)80135-3 11303975

[B20] Qiao X. Chen X. Wang W. Guo L. Pan Q. (2025). Classification of elderly patients with comorbidities and their subtypes: a data-driven cluster analysis. Clin. Interv. Aging 20, 1671–1680. 10.2147/cia.S549148 41063715 PMC12502974

[B21] Seta T. Noguchi Y. Shikata S. Nakayama T. (2014). Treatment of acute pancreatitis with protease inhibitors administered through intravenous infusion: an updated systematic review and meta-analysis. BMC Gastroenterol. 14, 102. 10.1186/1471-230x-14-102 24886242 PMC4061927

[B22] Singh V. P. Gorelick F. S. (2024). Has a hundred years of pursuing proteases helped to palliate pain in chronic pancreatitis more than placebo? Gastroenterology 166 (4), 559–561. 10.1053/j.gastro.2024.01.039 38311123

[B23] Sohail Z. Shaikh H. Iqbal N. Parkash O. (2024). Acute pancreatitis: a narrative review. J. Pak Med. Assoc. 74 (5), 953–958. 10.47391/jpma.9280 38783446

[B24] Song Y. Lee S. H. (2024). Recent treatment strategies for acute pancreatitis. J. Clin. Med. 13 (4), 978. 10.3390/jcm13040978 38398290 PMC10889262

[B25] Sundar V. Senthil Kumar K. A. Manickam V. Ramasamy T. (2020). Current trends in pharmacological approaches for treatment and management of acute pancreatitis - a review. J. Pharm. Pharmacol. 72 (6), 761–775. 10.1111/jphp.13229 32012276

[B26] Tenner S. Vege S. S. Sheth S. G. Sauer B. Yang A. Conwell D. L. (2024). American college of gastroenterology guidelines: management of acute pancreatitis. Am. J. Gastroenterol. 119 (3), 419–437. 10.14309/ajg.0000000000002645 38857482 PMC13221274

[B27] Trikudanathan G. Yazici C. Evans Phillips A. Forsmark C. E. (2024). Diagnosis and management of acute pancreatitis. Gastroenterology 167 (4), 673–688. 10.1053/j.gastro.2024.02.052 38759844

[B28] Wang R. Yang F. Wu H. Wang Y. Huang Z. Hu B. (2013). High-dose versus low-dose octreotide in the treatment of acute pancreatitis: a randomized controlled trial. Peptides 40, 57–64. 10.1016/j.peptides.2012.12.018 23275042

[B29] Wang G. Liu Y. Zhou S. F. Qiu P. Xu L. Wen P. (2016). Effect of somatostatin, ulinastatin and gabexate on the treatment of severe acute pancreatitis. Am. J. Med. Sci. 351 (5), 506–512. 10.1016/j.amjms.2016.03.013 27140710

[B30] Xie J. Li L. (2021). Efficacy of gabexate mesilate in preventing post endoscopic retrograde cholangiopancreatography pancreatitis: a meta-analysis of randomized clinical trials. J. Formos. Med. Assoc. 120 (9), 1796–1797. 10.1016/j.jfma.2021.03.034 33895063

[B31] Yang F. Wu H. Li Y. Li Z. Wang C. Yang J. (2012). Prevention of severe acute pancreatitis with octreotide in obese patients: a prospective multi-center randomized controlled trial. Pancreas 41 (8), 1206–1212. 10.1097/MPA.0b013e3182523bdf 23086244

[B32] Yasunaga H. Horiguchi H. Hashimoto H. Matsuda S. Fushimi K. (2013). Effect and cost of treatment for acute pancreatitis with or without gabexate mesylate: a propensity score analysis using a nationwide administrative database. Pancreas 42 (2), 260–264. 10.1097/MPA.0b013e31826495a0 23000890

[B33] Zhao M. Cui M. Fan M. Huang C. Wang J. Zeng Y. (2025). Octreotide attenuates experimental severe acute pancreatitis through inhibiting pyroptosis and modulating intestinal homeostasis. Eur. J. Pharmacol. 994, 177314. 10.1016/j.ejphar.2025.177314 39922420

[B34] Zou Z. Li Z. Wan Q. Wang Q. Yu Y. (2025). Association between a laboratory-based frailty index and mortality of critically ill patients with acute pancreatitis: a retrospective study. Front. Nutr. 12, 1519112. 10.3389/fnut.2025.1519112 40357041 PMC12066625

