# Supplementary material for: Adjunctive gabexate mesylate with octreotide and clinical outcomes in older adults with acute pancreatitis: a retrospective study
Source: Front Pharmacol. 2026 Oct 14;17:1755737. doi: 10.3389/fphar.2026.1755737 (PMC13713358; doi:10.3389/fphar.2026.1755737)
Supplement: Supplementary file 1 [file Supplementaryfile1.docx]

Supplementary Table S1. Detailed baseline characteristics of elderly patients with acute pancreatitis

| Variable | Combination therapy (n=108) | Octreotide monotherapy (n=112) | P value | Absolute SMD |
| --- | --- | --- | --- | --- |
| Demographic characteristics and comorbidities |  |  |  |  |
| Age, years | 71.9 ± 5.5 | 73.1 ± 5.7 | 0.113 | 0.214 |
| Male sex, n (%) | 60 (55.6) | 70 (62.5) | 0.295 | 0.142 |
| Body mass index, kg/m² | 24.6 ± 3.4 | 24.7 ± 3.2 | 0.823 | 0.030 |
| Hypertension, n (%) | 53 (49.1) | 61 (54.5) | 0.424 | 0.108 |
| Diabetes mellitus, n (%) | 35 (32.4) | 27 (24.1) | 0.171 | 0.185 |
| Coronary artery disease, n (%) | 10 (9.3) | 17 (15.2) | 0.181 | 0.181 |
| Chronic kidney disease, n (%) | 13 (12.0) | 8 (7.1) | 0.217 | 0.167 |
| Etiology |  |  | 0.205 | — |
| Biliary | 64 (59.3) | 51 (45.5) | — | 0.277 |
| Hypertriglyceridemia | 21 (19.4) | 31 (27.7) | — | 0.195 |
| Alcohol-related | 7 (6.5) | 7 (6.3) | — | 0.009 |
| Idiopathic | 16 (14.8) | 23 (20.5) | — | 0.150 |
| Revised Atlanta severity |  |  | 0.644 | — |
| Mild | 53 (49.1) | 62 (55.4) | — | 0.126 |
| Moderately severe | 38 (35.2) | 35 (31.3) | — | 0.084 |
| Severe | 17 (15.7) | 15 (13.4) | — | 0.067 |
| Admission clinical and laboratory indices |  |  |  |  |
| APACHE II score | 9.7 ± 2.8 | 9.1 ± 2.9 | 0.120 | 0.210 |
| C-reactive protein, mg/L | 125.4 ± 49.1 | 123.5 ± 52.4 | 0.782 | 0.037 |
| Procalcitonin, ng/mL | 0.89 ± 0.52 | 0.93 ± 0.49 | 0.558 | 0.079 |
| Serum amylase, U/L | 631.2 ± 306.6 | 647.0 ± 308.7 | 0.704 | 0.051 |
| Serum lipase, U/L | 937.6 ± 425.6 | 879.1 ± 434.3 | 0.314 | 0.136 |
| Blood urea nitrogen, mmol/L | 8.4 ± 3.1 | 7.8 ± 3.0 | 0.146 | 0.197 |
| Serum creatinine, μmol/L | 95.3 ± 26.4 | 89.7 ± 25.2 | 0.109 | 0.217 |
| SIRS on admission, n (%) | 48 (44.4) | 55 (49.1) | 0.488 | 0.094 |
| Time from symptom onset to treatment initiation, h | 19.7 ± 8.0 | 17.8 ± 8.6 | 0.091 | 0.229 |

Abbreviations: APACHE II, Acute Physiology and Chronic Health Evaluation II; SIRS, systemic inflammatory response syndrome; SMD, standardized mean difference.

Supplementary Table S2. Complete safety and complication outcomes in elderly patients with acute pancreatitis

| Outcome | Combination therapy (n=108) | Octreotide monotherapy (n=112) | Unadjusted OR | 95% CI | P value |
| --- | --- | --- | --- | --- | --- |
| New-onset or persistent SIRS after treatment initiation | 30 (27.8) | 46 (41.1) | 0.55 | 0.31 to 0.97 | 0.038 |
| Composite organ dysfunction | 19 (17.6) | 30 (26.8) | 0.58 | 0.31 to 1.12 | 0.101 |
| Respiratory failure | 8 (7.4) | 13 (11.6) | 0.61 | 0.24 to 1.53 | 0.289 |
| Renal impairment | 7 (6.5) | 12 (10.7) | 0.58 | 0.22 to 1.53 | 0.264 |
| Circulatory instability | 10 (9.3) | 17 (15.2) | 0.57 | 0.25 to 1.31 | 0.181 |
| Infectious complications | 15 (13.9) | 26 (23.2) | 0.53 | 0.26 to 1.07 | 0.076 |
| Invasive supportive interventions | 16 (14.8) | 27 (24.1) | 0.55 | 0.28 to 1.09 | 0.082 |
| In-hospital mortality | 3 (2.8) | 6 (5.4) | 0.5 | 0.12 to 2.07 | 0.499 |
| Drug-related adverse events | 6 (5.6) | 5 (4.5) | 1.26 | 0.37 to 4.25 | 0.765 |

P values were calculated using Pearson’s chi-square test, except for in-hospital mortality and drug-related adverse events, for which Fisher’s exact test was used.

Abbreviations: CI, confidence interval; OR, odds ratio; SIRS, systemic inflammatory response syndrome.

Supplementary Table S3. Specifications and analysis populations of the multivariable regression models

| Model | Outcome | Analysis population | Sample size/events | Regression model | Covariates entered |
| --- | --- | --- | --- | --- | --- |
| 1 | Time to abdominal pain relief | All included patients | n=220 | Multiple linear regression | Treatment regimen, age, sex, diabetes mellitus, chronic kidney disease, biliary etiology, APACHE II score, SIRS on admission, and time to treatment initiation |
| 2 | Time to normalization of pancreatic enzymes | All included patients | n=220 | Multiple linear regression | Treatment regimen, age, sex, diabetes mellitus, chronic kidney disease, biliary etiology, APACHE II score, SIRS on admission, and time to treatment initiation |
| 3 | Time to recovery of gastrointestinal function | All included patients | n=220 | Multiple linear regression | Treatment regimen, age, sex, diabetes mellitus, chronic kidney disease, biliary etiology, APACHE II score, SIRS on admission, and time to treatment initiation |
| 4 | Length of hospital stay | All included patients | n=220 | Multiple linear regression | Treatment regimen, age, sex, diabetes mellitus, chronic kidney disease, biliary etiology, APACHE II score, SIRS on admission, and time to treatment initiation |
| 5 | Duration of bowel rest | All included patients | n=220 | Multiple linear regression | Treatment regimen, age, sex, diabetes mellitus, chronic kidney disease, biliary etiology, APACHE II score, SIRS on admission, and time to treatment initiation |
| 6 | Duration of intravenous medication infusion | All included patients | n=220 | Multiple linear regression | Treatment regimen, age, sex, diabetes mellitus, chronic kidney disease, biliary etiology, APACHE II score, SIRS on admission, and time to treatment initiation |
| 7 | Radiological improvement | Patients undergoing repeat imaging | n=176; 124 events | Binary logistic regression | Treatment regimen, age, APACHE II score, revised Atlanta severity, biliary etiology, SIRS on admission, and time to treatment initiation |
| 8 | New-onset or persistent SIRS after treatment initiation | All included patients | n=220; 76 events | Binary logistic regression | Treatment regimen, age, APACHE II score, revised Atlanta severity, SIRS on admission, and time to treatment initiation |
| 9 | Infectious complications | All included patients | n=220; 41 events | Binary logistic regression | Treatment regimen, age, APACHE II score, SIRS on admission, and time to treatment initiation |
| 10 | Composite organ dysfunction | All included patients | n=220; 49 events | Binary logistic regression | Treatment regimen, age, APACHE II score, SIRS on admission, and time to treatment initiation |

Abbreviations: APACHE II, Acute Physiology and Chronic Health Evaluation II; SIRS, systemic inflammatory response syndrome.

Supplementary Table S4. Diagnostic performance and goodness-of-fit of the multivariable regression models

| Model | Adjusted R² or Nagelkerke R²* | Maximum VIF* | Calibration/assumption assessment* | Discrimination or residual assessment* |
| --- | --- | --- | --- | --- |
| 1 | Adjusted R² = 0.29 | 1.84 | Breusch–Pagan P=0.312 | Residual Q–Q plot acceptable |
| 2 | Adjusted R² = 0.34 | 1.86 | Breusch–Pagan P=0.228 | Residual Q–Q plot acceptable |
| 3 | Adjusted R² = 0.27 | 1.8 | Breusch–Pagan P=0.401 | Residual Q–Q plot acceptable |
| 4 | Adjusted R² = 0.25 | 1.92 | Breusch–Pagan P=0.174 | Residual Q–Q plot acceptable |
| 5 | Adjusted R² = 0.21 | 1.88 | Breusch–Pagan P=0.286 | Residual Q–Q plot acceptable |
| 6 | Adjusted R² = 0.18 | 1.75 | Breusch–Pagan P=0.355 | Residual Q–Q plot acceptable |
| 7 | Nagelkerke R² = 0.17 | 1.91 | Hosmer–Lemeshow P=0.624 | AUC 0.74 (95% CI 0.67–0.81) |
| 8 | Nagelkerke R² = 0.15 | 1.76 | Hosmer–Lemeshow P=0.581 | AUC 0.72 (95% CI 0.65–0.79) |
| 9 | Nagelkerke R² = 0.13 | 1.68 | Hosmer–Lemeshow P=0.713 | AUC 0.71 (95% CI 0.63–0.79) |
| 10 | Nagelkerke R² = 0.12 | 1.7 | Hosmer–Lemeshow P=0.646 | AUC 0.70 (95% CI 0.62–0.78) |

Abbreviations: AUC, area under the receiver operating characteristic curve; CI, confidence interval; Q–Q, quantile–quantile; R², coefficient of determination; VIF, variance inflation factor.

Supplementary Table S5. Treatment associations stratified by acute pancreatitis severity according to the revised Atlanta classification

| Outcome | Mild AP, combination/monotherapy n=53/62 | Moderately severe AP, combination/monotherapy n=38/35 | Severe AP, combination/monotherapy n=17/15 | P for interaction |
| --- | --- | --- | --- | --- |
| Continuous outcomes, adjusted mean difference (95% CI), days |  |  |  |  |
| Time to abdominal pain relief | −0.88 (−1.25 to −0.51) | −0.98 (−1.50 to −0.46) | −1.12 (−2.05 to −0.19) | 0.83 |
| Time to normalization of serum pancreatic enzymes | −1.18 (−1.62 to −0.74) | −1.31 (−1.95 to −0.67) | −1.42 (−2.56 to −0.28) | 0.79 |
| Time to recovery of gastrointestinal function | −0.79 (−1.23 to −0.35) | −0.90 (−1.52 to −0.28) | −1.10 (−2.06 to −0.14) | 0.87 |
| Length of hospital stay | −1.20 (−2.02 to −0.38) | −1.73 (−2.97 to −0.49) | −2.24 (−4.18 to −0.30) | 0.61 |
| Binary outcome, combination therapy vs monotherapy, n/N (%) |  |  |  |  |

Abbreviations: AP, acute pancreatitis; CI, confidence interval.

Supplementary Table S6. Thirty- and 90-day mortality and readmission outcomes according to treatment group

| Outcome | Analysis population | Combination therapy, n/N (%) | Octreotide monotherapy, n/N (%) | Firth-adjusted OR | 95% CI |
| --- | --- | --- | --- | --- | --- |
| 30-day all-cause mortality from index admission | All included patients | 4/108 (3.7) | 8/112 (7.1) | 0.52 | 0.14 to 1.92 |
| 90-day all-cause mortality from index admission | All included patients | 6/108 (5.6) | 11/112 (9.8) | 0.58 | 0.20 to 1.68 |
| 30-day all-cause institutional readmission after discharge | Patients discharged alive, alive at 30 days, and with ascertainable readmission status | 10/103 (9.7) | 17/104 (16.3) | 0.59 | 0.25 to 1.39 |

Abbreviations: CI, confidence interval; OR, odds ratio.
